# Atypical chemokine receptor D6 inhibits human non-small cell lung cancer growth by sequestration of chemokines

**DOI:** 10.3892/ol.2013.1358

**Published:** 2013-05-21

**Authors:** FENG YING WU, JIANG FAN, LIANG TANG, YIN MIN ZHAO, CAI CUN ZHOU

**Affiliations:** 1 Central Laboratory of Oncology, Tongji University School of Medicine, Shanghai 200433, P.R. China; 2 Department of Thoracic Surgery, Shanghai Pulmonary Hospital, Tongji University School of Medicine, Shanghai 200433, P.R. China

**Keywords:** chemokines, D6, decoy receptor, lung neoplasm, proliferation

## Abstract

Chemokines and their receptors have been shown to play a vital role in lung cancer progression. D6 is an atypical chemokine receptor which is able to internalize and degrade chemokines. To investigate the potential role of D6 in lung cancer, we established D6-overexpressing A549 lung cancer cell lines by the transfection of human D6 cDNA. Results showed that D6 inhibited the proliferation of cancer cells *in vitro* and tumorigenesis *in vivo*. We also determined chemokine levels in the supernatant and showed that a number of chemokines (CCL2/4/5) had significantly decreased protein levels in D6-overexpressing cells compared with the controls, whereas no significant changes in mRNA expression levels of these chemokines were detected. The cell cycle distribution and expression of certain growth factors and their receptors did not change in the D6-overexpressing cells compared with parental cells. Thus, our results suggest that D6 is a negative regulator of growth in lung cancer, mainly by the sequestration of specific chemokines.

## Introduction

Lung cancer is the leading cause of cancer-associated mortality worldwide. Efforts at improving the poor prognosis of patients with lung cancer partially depend upon a better understanding of the biology of lung cancer. Chemokines are multifunctional small peptides which recruit and activate subpopulations of leukocytes, in addition to numerous other cell types. They are classified into four groups (CC, CXC, CX3C and XC), according to their characteristic cysteine motifs. Chemokines and their receptors have been identified as playing a vital role in tumor progression, where they act as growth factors, increase angiogenesis, subvert immunological surveillance and induce metastasis to distant sites (1–3).

Atypical chemokine receptors comprise a three-member subfamily of chemokine receptors: Duffy antigen receptor for chemokines (DARC), D6 and ChemocentryX chemokine receptor (CCX-CKR). These receptors differ from other chemokine receptors as they efficiently internalize their cognate chemokine ligands and act as chemokine scavengers. To date, there are no known signaling cascades activated by them. It is suggested that they act as decoy receptors to compete for ligand binding or to control the availability of ligands in a particular environment (4). In our previous study, we showed that DARC, D6 and CCX-CKR have inhibitory roles in breast cancer by sequestering chemokines individually (5–7). In lung cancer, Addison *et al* observed that DARC-expressing A549 cells have a significant reduction in cellularity, increased levels of necrosis, lower microvessel density and decreased metastasis (8). However, the role of D6 in lung cancer has not been clearly defined. To further investigate the role of D6 in lung cancer and the possible mechanisms involved, we induced the overexpression of D6 in lung cancer cell lines and results showed that D6 inhibited the proliferation of lung cancer cells. Furthermore, this inhibitory effect was coupled with the clear degradation of chemokines in the cell supernatant.

## Materials and methods

### Cell lines and reagents

The A549 human lung adenocarcinoma cell line was obtained from the ATCC (American Type Culture Collection, Manassas, VA, USA) and cultured according to the manufacturer’s instructions. An RPMI-1640 medium (Invitrogen Life Technologies, Inc., Carlsbad, CA, USA) containing 10% fetal bovine serum (Invitrogen Life Technologies, Inc.) was used as a culture medium. Real-time PCR reagents were obtained from Takara Bio, Inc. (Shiga, Japan). Rat anti-human D6 monoclonal antibody was purchased from R&D Systems (Minneapolis, MN, USA). The plasmid pcDNA3.0/D6 was kindly provided by Professor Albert Mantovani (Istituto Clinico Humanitas, University of Milan, Milan, Italy). The study was approved by the Ethics Committee of Shanghai Pulmonary Hospital, Tongji University School of Medicine, Shanghai 200433, China.

### Generation of stable transfected non-small cell lung cancer cell lines

The whole cDNA sequence of D6 was obtained from the pcDNA3.0/D6 plasmid by PCR and was reconstructed into a lentiviral vector PLenti-GFP-Neo (PLenti-GFP-Neo/D6), which was obtained from Shanghai Telebio Biomedical Co., Ltd., Shanghai, China. The vector contained a neomycin-resistant gene for establishing a stable cell line and a coral GFP gene for tracking transfection efficiency. It also uses a Rous sarcoma virus (RSV) enhancer/promoter joined to HIV 5′LTR and HIV 3′LTR for viral transcription and packaging. Cells were cultured in 6-well plates and infected with virus vectors when the monolayers had reached 40–60% confluence. As a standard procedure, monolayers were washed twice with medium lacking fetal calf serum (FCS; washing medium) and overlaid with washing medium containing a lentivirus with a multiplicity of infection (MOI) of 50. After incubation overnight at 37°C, any non-adsorbed virus was removed and a medium containing FCS was added for incubation over 48 h at 37°C (9). Fluorescence microscopy was used to detect transduced cells exhibiting GFP-induced fluorescence. Following transfection, cells were grown in the presence of G418 and G418-resistant colonies were isolated and expanded in culture. Two independently generated D6-overexpressing A549 cell lines were selected for further analysis; A549/D6-1 and A549/D6-2. The D6-positive colonies were further identified by RT-PCR and western blotting.

### RNA extraction and reverse transcription-PCR

Total RNA was isolated from cells using TRIzol reagent (Invitrogen, Carlsbad, CA, USA) according to the manufacturer’s instructions. The specific primers of D6 and other relevant molecules used in this study are listed in Table I. All experiments were repeated in triplicate.

### Proliferation assay and cell cycle analysis

Cell proliferation was carried out using Cell Counting Kit-8 (CCK-8, Dojindo, Kunmamoto, Japan) according to the manufacturer’s instructions. Flow cytometry analysis of the DNA content was carried out to assess the cell cycle phase distribution.

### Western blot analysis

Cells were washed twice with ice-cold phosphate-buffered saline (PBS) and scraped into 1.0 ml of ice-cold NP40 lysis buffer. Cells were then sonicated for 5 sec at 5 W. Insoluble debris was removed by centrifugation at 12,000 rpm for 30 min. Total proteins (50 μg) were analyzed by 10% SDS-PAGE. Western blotting with rat anti-human D6 monoclonal antibody was carried out according to standard procedures. Blot quantification was carried out using a Molecular Dynamics Laser Densitometer and the ImageQuant version 1 software (Molecular Dynamics, San Jose, CA, USA).

### ELISA

Chemokine levels in the cell culture medium were measured using the human ELISA Ready-SET-Go kit (R&D Systems, Minneapolis, MN, USA) according to manufacturer’s instructions. The absorbance of the test sample was compared with the standard curve. The concentrations were determined in duplicate according to the manufacturer’s instructions.

### Statistical analysis

Statistical analysis was conducted using the SPSS software. ANOVA and Student’s t-test were used to determine the statistical significance of differences between the experimental groups. P<0.05 was considered to indicate a statistically significant difference.

## Results

### D6 inhibits the proliferation of cells in vitro

**Figure 1.**
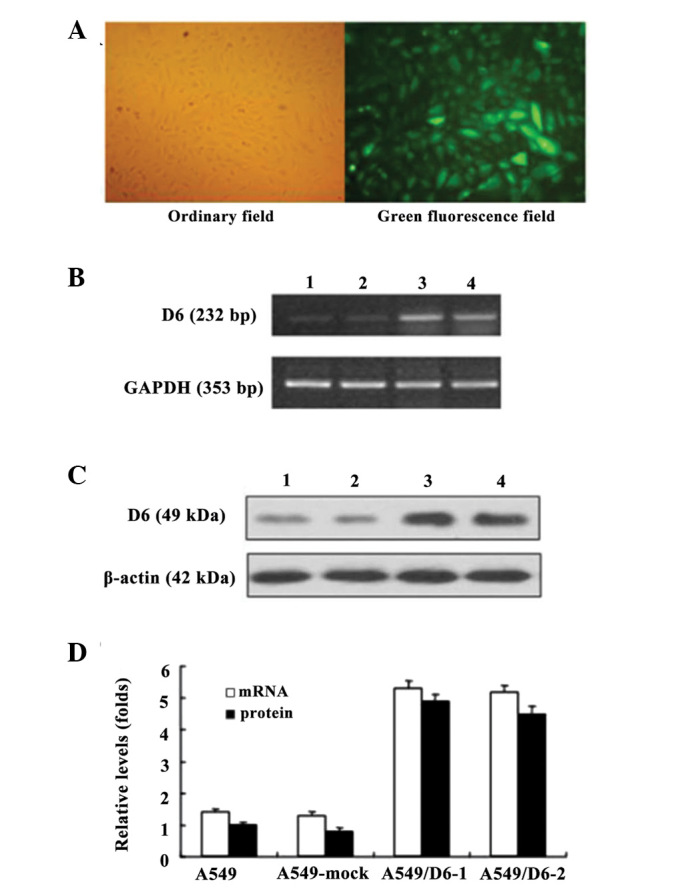
Stable transduction of D6 into A549 lung cancer cells. (A) Microscopy analysis of A549 cells transfected with Lenti-D6 just before G418 selection. (B and C) Expression of D6 in A549 cells after Lenti-D6 transfection was detected by RT-PCR and western blotting. Lane 1, A549; lane 2, A549-mock; lane 3, A549/D6-1; and lane 4, A549/D6-2. (D) Relative mRNA expression and protein levels in A549/D6-1 and A549/D6-2 clones.

**Figure 2.**
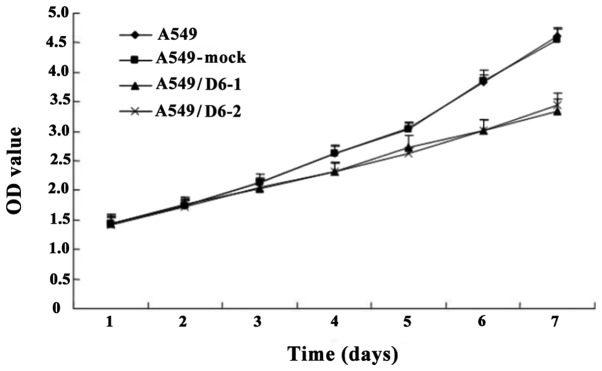
Growth curve of A549, A549-mock, A549/D6-1 and A549/D6-2 for the proliferation assay. D6 inhibits the growth of A549 cells. P<0.05 vs. A549 and A549-mock cell lines; bars, SE.

D6 was constitutively expressed by A549 lung cancer cells. We successfully generated D6-overexpressing A549 cell lines by stable transfection. The transfection efficiency was initially evaluated using immunofluorescence. After selection, stable D6-overexpressing clones were further confirmed by RT-PCR and western blotting (Fig. 1). To explore whether D6 was able to modulate proliferation *in vitro*, we conducted a proliferation assay. Compared with mock-transfected and parental cells, D6-overexpressing cell growth was significantly slower, with the proliferative ability of cells being inhibited by 22% at day 4 (Fig. 2; P<0.05). Furthermore, flow cytometry analysis showed that cell cycle distribution changed in the D6-overexpressing clones, with the population of cells in G0/G1 phase showing an increase (66% in A549/D6 vs. 61.5% in controls); however, this difference was not statistically significant (data not shown).

### D6 enhances the clearance of chemokines in the cell super-natant

**Figure 3.**
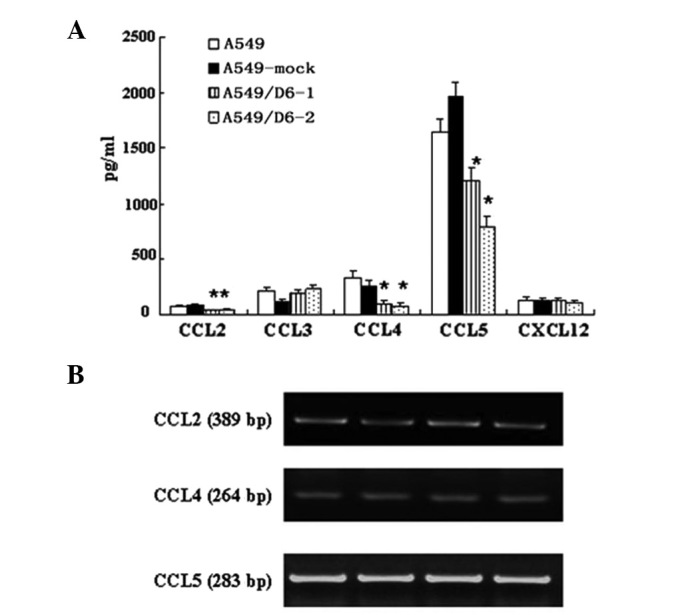
(A) Median levels of human chemokine levels in cell supernatant. Columns, mean of three independent experiments; bars, SE. ^*^P<0.05. (B) mRNA expression levels of CCL2, 4 and 5.

Since D6 is known to bind and scavenge its cognate chemokines, we detected the amount of these ligands (CCL2/3/4/5 and CXCL12) in cell supernatants. Results showed that a few of these ligands (CCL2/4/5) decreased significantly in the supernatant of D6-overexpressing cell lines; however, no clear change in their mRNA expression levels was detected (Fig. 3). Moreover, the mRNA expression levels of epidermal growth factor (EGF), basic fibroblast growth factor (bFGF), transforming growth factor-β (TGF-β) and epidermal growth factor receptor (EGFR) showed no change in D6-overexpressing clones compared with parental cells (data not shown).

### D6 inhibits tumorigenesis in BALB/c mice

**Figure 4.**
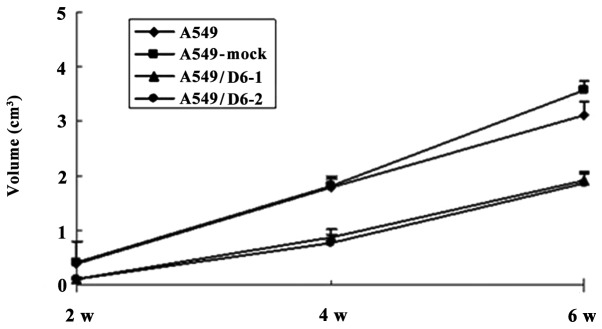
Growth curve of the *in vivo* proliferation assay.

To further explore the role of D6 *in vivo*, we used orthotopic xenograft tumor models in nude mice. As hypothesized, D6-overexpressing cell growth was slower than parental cells in the nude mice. We observed a 40% decrease over 40 days in A549/D6 (1885.3±160 mm^3^) tumor volumes compared with those of the mock-transfected (3105.3±172 mm^3^) and wild-type (3575.3±254 mm^3^) A549 tumors (P<0.05). The weight of xenograft tumors was also measured and the D6-overexpressing cell lines showed clear reductions in weight compared with the control tumors (Fig. 4; P<0.05).

## Discussion

In the tumor microenvironment, cancer, stromal and infiltrated immune cells may secrete chemokines. Cancer cells constitutively express chemokine receptors which respond to these chemokines. Chemokine ligand-receptor interactions form a complex network which affects tumor progression (10,11). A group of receptors, known as decoy receptors, have been identified in the chemokine system; DARC, D6 and CCX-CKR. It has been proposed that these atypical receptors may work in multiple ways, by competing for ligand binding and hence inhibiting the migration of cells bearing typical receptors and by internalizing and degrading ligands and therefore depleting chemokine levels in a particular micro-environment (4). Therefore, these atypical receptors may be involved in cancer progression by affecting chemokine levels in the tumor microenvironment.

D6, which is known to act as a decoy receptor, is able to bind and degrade the majority of inflammatory CC chemokines (CCL2/3L1/4/5/7/8/11/12/13/14/22 and weakly to CCL17) (12,13). The majority of these chemokines have been linked with various aspects of cancer biology. Considering that D6 functions as a decoy receptor by clearing these ligands, we hypothesized that D6 may also be involved in tumor progression by regulating chemokine levels, and existing studies have confirmed this hypothesis; Nibbs *et al* observed that D6 inhibits chemically induced skin *de novo* tumorigenesis via sequestration of inflammatory CC chemokines (14). Additionally, our previous study showed that D6 had an inhibitory role in breast cancer growth and metastasis and was negatively correlated with prognosis (7). However, there is little evidence showing the role of D6 in other tumor types, for example in lung cancer. In the present study, we successfully established D6-overexpressing A549 lung cancer cell lines A549/D6-1 and A549/D6-2 by stable transfection and demonstrated that D6 inhibited tumor growth *in vitro* and *in vivo*.

Accumulating results from previous studies suggest that chemokines may contribute to tumor cell growth directly, by acting as growth factors, or indirectly, through other signal pathways. Recombinant human CCL2 induces dose-dependent prostate cancer cell proliferation *in vitro* and this pro-growth function is accomplished by the activation of the phosphatidylinositol 3-kinase (PI3K)/AKT pathway (15,16). Additionally, PI3K/AKT activation by CCL2 mediates mTORC1 activation, survivin upregulation and subsequent downregulation of autophagosome formation, which provides a survival advantage (17). CCL4 was significantly overexpressed in mantle cell lymphomas cells compared with B cells, which may provide growth benefits (18). CCL5 activation stimulates the growth of MCF-7 breast cancer cells through an mTOR-dependent mechanism. Specifically, CCL5 mediates a rapid upregulation of protein expression for cyclin D1, c-Myc and Dad-1 (19). Another chemokine, CXCL12, was able to induce auto-/paracrine cell proliferation in human pituitary adenomas (20). In the present study, we demonstrated that the protein levels of ligands CCL2, 4 and 5 decreased significantly in the medium of D6-overexpressing cells compared with that of parental cells, whereas the expression of various growth factors and growth factor receptors showed no change, suggesting these chemokines may regulate A549 lung cancer cell growth directly. The mRNA expression levels of chemokines whose protein levels were decreased exhibited no change when compared with parental cells, suggesting that D6 modulates chemokines via post-translational pathways.

A previous study demonstrated that the depletion of certain chemokines was able to lead to cell cycle arrest at the G1/S boundary (21). In this study, we identified that the fraction of cells in G0/G1 phase in D6-overexpressing cells appeared to be higher than that of control cells; however, this result failed to reach statistical significance.

It is widely accepted that EGF, vascular endothelial growth factor, bFGF and EGFR are correlated with cancer cell growth. Thus, we compared the mRNA expression levels of these molecules and no clear differences were identified between D6-overexpressing cell lines and parental cells, suggesting that D6 has no effect on the expression of these molecules.

Our results indicate that the overexpression of the atypical chemokine receptor D6 attenuates lung cancer cell growth by post-translational clearing of chemokines. However, to better understand the regulation of D6 in a chemotactic network in human tumors, further investigation with regard to the regulation of D6 expression in tumors, D6 expression as a prognostic factor in lung cancer patients, the effects of D6 on sensitivity to chemotherapy and targeted therapy and the potential of D6 as a new therapeutic avenue for cancer treatment is required. Data from the current study provide primary evidence for further exploration of the role and therapeutic potential of D6 in cancer.

## Figures and Tables

**Table I.** Primers for RT-PCR and annealing temperatures for individual genes.

Gene	Primer sequence	Size of product (bp)	Temperature (°C)
D6	Upstream 5′-CCT GCT CCT TGC TAC CAT AGT AGT G-3′	232	60
	Downstream 5′-CAC CAA GAC ACA ACC AAT ACG GGA G-3′		
GAPDH	Upstream 5′-GGG AGC CAA AAG GGT CAT CAT CTC-3′	353	60
	Downstream 5′-CCA TGC CAG TGA GCT TCC CGT TC-3′		
CCL2	Upstream 5′-ACT GAA GCT CGC ACT CTC GCC TC-3′	389	60
	Downstream 5′-TGT CTG GGG AAA GCT AGG GGA AAA T-3′		
CCL4	Upstream 5′-TGT CCT GTC TCT CCT CAT GCT AGT A-3′	264	60
	Downstream 5′-GCT CAG TTC AGT TCC AGG TCA TAC A-3′		
CCL5	Upstream 5′-AAG GTC TCC GCG GCA CGC CTC-3′	283	60
	Downstream 5′-ACT CTC CAT CCT AGC TCA TCT CCA AA-3′		
EGF	Upstream 5′-GAC AAC TCC CCT AAG GCT TA-3′	462	55
	Downstream 5′-CAT GCA CAC GCC ACC ATT GAG GCA GTA CCC ATC GTA CGA-3′	566	
bFGF	Upstream 5′-TTC CCA CCAGGC CAC TTCA-3′	212	48
	Downstream 5′-GCC GTC CAT CTT CCT TCA TAG C-3′		
TGF-β	Upstream 5′-GAC TCC TGC TGC TTT CTC C-3′	531	60
	Downstream 5′-GCG GTC CAC CAT TAG CAC-3′		
EGFR	Upstream 5′-CTT CTT GCA GCG ATA CAG CTC-3′	440	60
	Downstream 5′-ATG CTC CAA TAA ATT CAC TGC-3′		

Bp, base pair; EGFR, epidermal growth factor receptor; bFGF, basic fibroblast growth factor; TGF-β, transforming growth factor-β.

## References

[1] Mantovani A, Savino B, Locati M, Zammataro L, Allavena P, Bonecchi R (2010). The chemokine system in cancer biology and therapy. Cytokine Growth Factor Rev.

[2] Zlotnik A (2006). Chemokines and cancer. Int J Cancer.

[3] Ben-Baruch A (2006). The multifaceted roles of chemokines in malignancy. Cancer Metastasis Rev.

[4] Comerford I, Litchfield W, Harata-Lee Y, Nibbs RJ, McColl SR (2007). Regulation of chemotactic networks by ‘atypical’ receptors. Bioessays.

[5] Feng LY, Ou ZL, Wu FY, Shen ZZ, Shao ZM (2009). Involvement of a novel chemokine decoy receptor CCX-CKR in breast cancer growth, metastasis and patient survival. Clin Cancer Res.

[6] Wang J, Ou ZL, Hou YF (2006). Enhanced expression of Duffy antigen receptor for chemokines by breast cancer cells attenuates growth and metastasis potential. Oncogene.

[7] Wu FY, Ou ZL, Feng LY (2008). Chemokine decoy receptor d6 plays a negative role in human breast cancer. Mol Cancer Res.

[8] Addison CL, Belperio JA, Burdick MD, Strieter RM (2004). Overexpression of the duffy antigen receptor for chemokines (DARC) by NSCLC tumor cells results in increased tumor necrosis. BMC Cancer.

[9] Ramezani A, Hawley RG (2002). Overview of the HIV-1 Lentiviral Vector System.

[10] Kruizinga RC, Bestebroer J, Berghuis P (2009). Role of chemokines and their receptors in cancer. Curr Pharm Des.

[11] Wong D, Korz W (2008). Translating an Antagonist of Chemokine Receptor CXCR4: from bench to bedside. Clin Cancer Res.

[12] Graham GJ, McKimmie CS (2006). Chemokine scavenging by D6: a movable feast?. Trends Immunol.

[13] Locati M, Torre YM, Galliera E (2005). Silent chemoattractant receptors: D6 as a decoy and scavenger receptor for inflammatory CC chemokines. Cytokine Growth Factor Rev.

[14] Nibbs RJ, Gilchrist DS, King V (2007). The atypical chemokine receptor D6 suppresses the development of chemically induced skin tumors. J Clin Invest.

[15] Loberg RD, Day LL, Harwood J (2006). CCL2 is a potent regulator of prostate cancer cell migration and proliferation. Neoplasia.

[16] Lu Y, Cai Z, Galson DL (2006). Monocyte chemotactic protein-1 (MCP-1) acts as a paracrine and autocrine factor for prostate cancer growth and invasion. Prostate.

[17] Roca H, Varsos Z, Pienta KJ (2008). CCL2 protects prostate cancer PC3 cells from autophagic death via phosphatidylinositol 3-kinase/AKT-dependent survivin up-regulation. J Biol Chem.

[18] Ek S, Björck E, Högerkorp CM (2006). Mantle cell lymphomas acquire increased expression of CCL4, CCL5 and 4-1BB-L implicated in cell survival. Int J Cancer.

[19] Murooka TT, Rahbar R, Fish EN (2009). CCL5 promotes proliferation of MCF-7 cells through mTOR-dependent mRNA translation. Biochem Biophys Res Commun.

[20] Barbieri F, Bajetto A, Stumm R (2008). Overexpression of stromal cell-derived factor 1 and its receptor CXCR4 induces autocrine/paracrine cell proliferation in human pituitary adenomas. Clin Cancer Res.

[21] Singh RK, Lokeshwar BL (2009). Depletion of intrinsic expression of Interleukin-8 in prostate cancer cells causes cell cycle arrest, spontaneous apoptosis and increases the efficacy of chemotherapeutic drugs. Mol Cancer.

